# Microbial and metabolomic profiles of type 1 diabetes with depression: A case–control study

**DOI:** 10.1111/1753-0407.13542

**Published:** 2024-04-10

**Authors:** Ziyu Liu, Tong Yue, Xueying Zheng, Sihui Luo, Wen Xu, Jinhua Yan, Jianping Weng, Daizhi Yang, Chaofan Wang

**Affiliations:** ^1^ Department of Endocrinology and Metabolism The Third Affiliated Hospital of Sun Yat‐sen University, Guangdong Diabetes Prevention and Control Research Center, Guangdong Provincial Key Laboratory of Diabetology Guangzhou China; ^2^ Department of Endocrinology The Sixth Affiliated Hospital of Sun Yat‐sen University Guangzhou China; ^3^ Department of Endocrinology, Institute of Endocrine and Metabolic Diseases The First Affiliated Hospital of USTC, Division of Life Sciences and Medicine, Clinical Research Hospital of the Chinese Academy of Sciences (Hefei), University of Science and Technology of China Hefei China

**Keywords:** depression, metabolomics, microbiomics, type 1 diabetes

## Abstract

**Background:**

Depression is the most common psychological disorder in patients with type 1 diabetes (T1D). However, the characteristics of microbiota and metabolites in these patients remain unclear. This study aimed to investigate microbial and metabolomic profiles and identify novel biomarkers for T1D with depression.

**Methods:**

A case–control study was conducted in a total of 37 T1D patients with depression (TD+), 35 T1D patients without depression (TD−), and 29 healthy controls (HCs). 16S rRNA gene sequencing and liquid chromatography–mass spectrometry (LC–MS) metabolomics analysis were conducted to investigate the characteristics of microbiota and metabolites. The association between altered microbiota and metabolites was explored by Spearman's rank correlation and visualized by a heatmap. The microbial signatures to discriminate TD+ from TD− were identified by a random forest (RF) classifying model.

**Results:**

In microbiota, 15 genera enriched in TD− and 2 genera enriched in TD+, and in metabolites, 14 differential metabolites (11 upregulated and 3 downregulated) in TD+ versus TD− were identified. Additionally, 5 genera (including *Phascolarctobacterium*, *Butyricimonas*, and *Alistipes* from altered microbiota) demonstrated good diagnostic power (area under the curve [AUC] = 0.73; 95% CI, 0.58–0.87). In the correlation analysis, *Butyricimonas* was negatively correlated with glutaric acid (*r* = −0.28, *p* = 0.015) and malondialdehyde (*r* = −0.30, *p* = 0.012). Both *Phascolarctobacterium* (*r* = 0.27, *p* = 0.022) and *Alistipes* (*r* = 0.31, *p* = 0.009) were positively correlated with allopregnanolone.

**Conclusions:**

T1D patients with depression were characterized by unique profiles of gut microbiota and serum metabolites. *Phascolarctobacterium*, *Butyricimonas*, and *Alistipes* could predict the risk of T1D with depression. These findings provide further evidence that the microbiota–gut–brain axis is involved in T1D with depression.

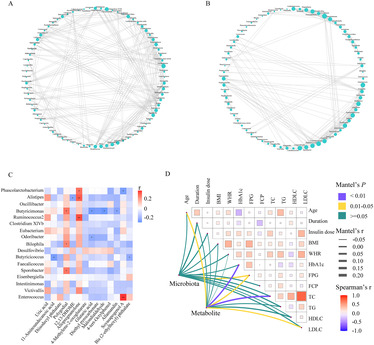

## Graphical abstract

Highlights

- Type 1 diabetes (T1D) patients with depression were characterized by unique profiles of gut microbiota and serum metabolites.
- *Phascolarctobacterium*, *Butyricimonas*, and *Alistipes* could predict the risk of T1D with depression.

## 1 INTRODUCTION

Type 1 diabetes (T1D), an autoimmune disease characterized by the destruction of insulin‐producing β cells in the pancreas, poses a significant health challenge worldwide.^1^ Individuals with T1D have an increased risk of psychological disorders including depression, anxiety, eating disorder, and substance abuse.^2^ These mental disorders in diabetes are associated with poor compliance with treatment and inadequate glycemic control, which increases the risk for severe diabetic complications and represents a major medical and economic burden worldwide.^3^, ^4^, ^5^

Depression is the most common psychological disorder in patients with T1D.^6^ A previous meta‐analysis including 14 studies showed that the pooled prevalence of depressive symptoms was 30% in children and adolescents with T1D,^7^ and another study reported that the prevalence of depression was 24% in adults with T1D.^6^ However, the pathogenesis of depression in T1D is still unclear. Biological risk factors affecting the central nervous system are likely to be involved.^8^, ^9^

It has been reported that significant differences have been observed in the composition of gut microbiota and their associated metabolites in patients with T1D.^10^ Patients with T1D exhibit compositional alterations in the gut microbiota, including a decreased ratio of Firmicutes to *Bacteroides*, a decreased abundance of short‐chain fatty acid‐producing bacteria, and an increased abundance of *Bacteroides* and *Bifidobacterium*.^11^ However, the alteration of *Bifidobacterium* and *Streptococcus* lacks consistency.^12^, ^13^, ^14^ It was reported that gut microbiota might influence the psychological state via the microbiota–gut–brain axis.^15^, ^16^, ^17^ Similarly, studies on the effects of probiotic or prebiotic intake indicated an important role for the microbiota in regulating depression, anxiety, and other emotional responses.^18^, ^19^, ^20^ In addition, metabolomic tools contributed to a better understanding of the role of gut microbiota in the development of mental disorders.^21^, ^22^ While the common biological mechanisms of type 2 diabetes and depression (including insulin resistance, hippocampal atrophy, and endothelial dysfunction) are becoming increasingly understood,^23^, ^24^ the corresponding evidence for T1D is still limited^25^ and the correlations between microbial and metabolomic changes in T1D are currently rarely discussed.^26^, ^27^

In this study, we aimed to investigate microbial and metabolomic profiles and identify novel biomarkers for T1D with depression.

## 2 MATERIALS AND METHODS

### 2.1 Study populations

**FIGURE 1:**
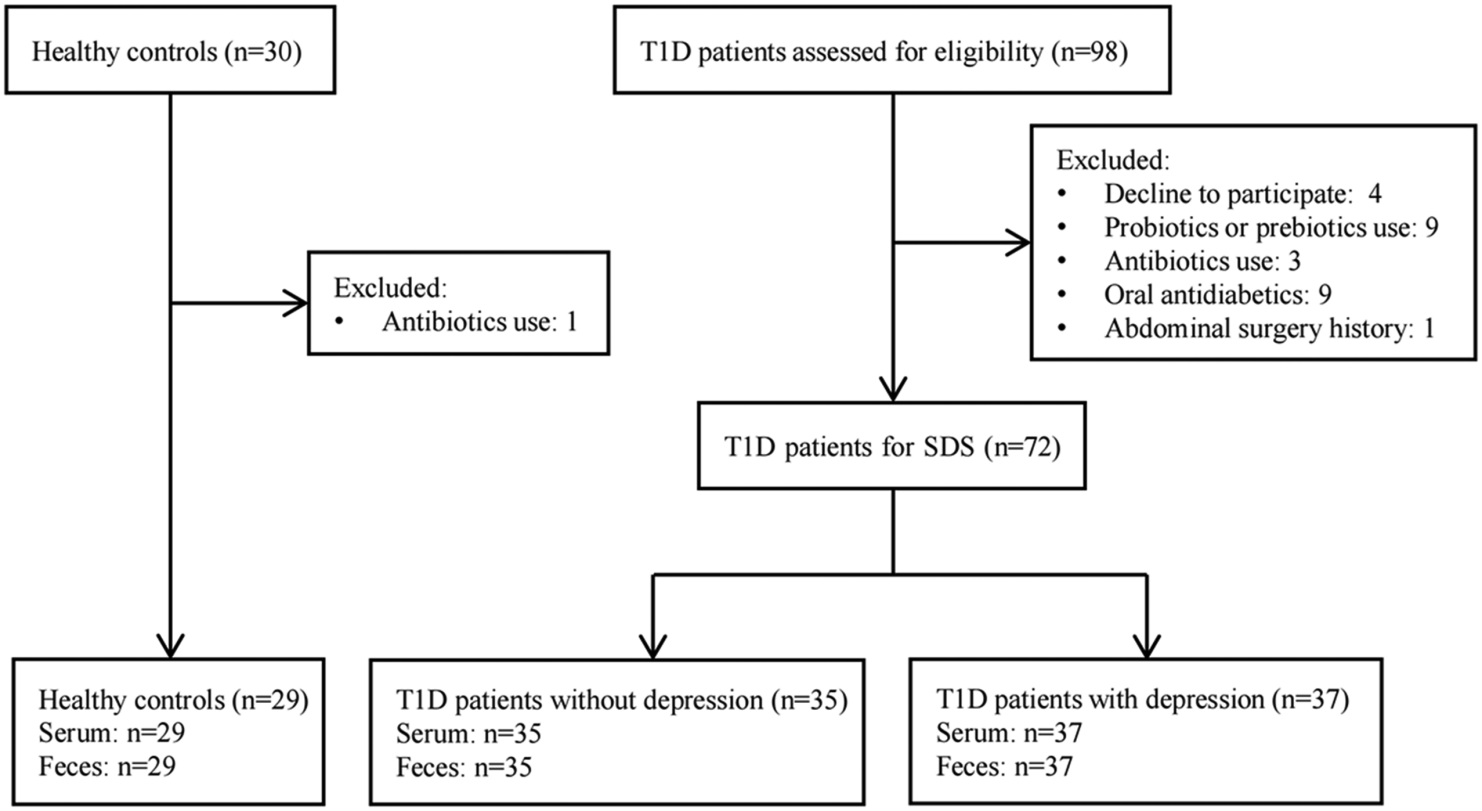
The flow chart of study participant selection. SDS, self‐rating depression scale; T1D, type 1 diabetes.

This study was designed as a noninterventional cross‐sectional case–control study. All subjects were enrolled at the Third Affiliated Hospital of Sun Yat‐sen University, Guangzhou, China. T1D patients were participants of the T1D China Registry Study (www.chictr.org.cn, ChiCTR2000034642).^28^ Inclusion criteria were participants diagnosed with T1D based on previous studies^28^, ^29^ and were at least 18 years of age. Exclusion criteria were (1) acute or chronic gastrointestinal disease; (2) history of intestinal surgery; (3) dietary modulation that was known to affect the microbiota; (4) use of antibiotics in the previous 3 months; (5) use of probiotics, prebiotics, or synbiotics in the previous month; or (6) combined neurological illness confirmed by a detailed clinical assessment. The recruitment of participants is depicted in Figure 1.

We consecutively recruited 72 T1D patients, including 37 T1D patients with depression (TD+) and 35 T1D patients without depression (TD−), and 29 healthy controls (HCs) matched for age and gender were recruited through public advertisements. The protocol for this study conformed to the ethical guidelines of the Declaration of Helsinki and was approved by the ethics committee of the Third Affiliated Hospital of Sun Yat‐sen University (approval no. [2014]2‐1051). All participants provided informed written consent.

### 2.2 Psychological assessment

The self‐rating depression scale (SDS) was used to assess the depression of all participants.^30^ The scale included 20 items, with each item scoring from 1 to 4. It was calculated as the sum scores of all the items and then multiplied by a weight of 1.25. A higher score suggests a more sever status of depression, and a score ≥53 suggests depression in the Chinese population.^31^ The assessment training for researchers was conducted to ensure consistency. After assessment, patients with an SDS score ≥53 were further diagnosed by psychologists based on the *International Classification of Diseases, Tenth Revision* (ICD‐10) criteria.

### 2.3 Clinical data and sample collection

Anthropometric and clinical data were collected from all participants, and T1D patients were instructed to follow healthy dietary patterns for diabetes. For microbiota and metabolomics analysis, fresh feces and serum samples were collected from the study population and kept in sterile tubes. All the samples were stored at −80°C as soon as possible till processing.

### 2.4 Fecal DNA extraction and 16S rRNA sequencing

The fecal DNA was extracted by the MagPure Stool DNA KF Kit B (Magen, China) according to the manufacturer's instructions. 16S rRNA gene amplification was performed by directional primers (515F: 5′‐ACTCCTACGGGAGGCAGCAG‐3′; 806R: 5′‐GGACTACHVGGGTWTCTAAT‐3′) targeting the V4 hypervariable regions. The polymerase chain reaction (PCR) was performed in triplicate, each reaction comprising a volume of 50 μL. The PCR products were purified using AmpureXP beads and eluted in the elution buffer. The validated libraries were used for sequencing on the Illumina MiSeq platform (BGI, Shenzhen, China) and generating 2 × 250 bp paired‐end reads.

### 2.5 Sequencing data processing

Fastq file analysis was performed using the Quantitative Insights Into Microbial Ecology (QIIME2).^32^ Reads of 250 bp were clipped for each consecutive three‐locus position with an average quality score of 20. Paired‐end reads were added to tags by the Fast Length Adjustment of Short Reads (FLASH, 1.2.11).^33^ Tags were classified as operational tax units (OTUs) by UPARSE 7.0.1090 with a cutoff of 97%, and UCHIME 4.2.40 was used to verify and remove chimeric sequences for further analysis. The remaining high‐quality sequences were grouped into operationally amplified sequence variants (ASVs) by the DADA2 algorithm with 100% similarity.^34^ Using the Ribosomal Database Project Classifier (RDP 11.5), representative ASVs were annotated with taxonomic information based on the Mothur algorithm.

### 2.6 Metabolite extraction and data processing

After being thawed slowly at 4°C, 300 μL of precooled methanol and acetonitrile (2:1, v:v) were added for extracting 100‐μL samples, and quality control was performed by Internal Standards Mix 1 (IS1) and Internal Standards Mix 2 (IS2). After a 1‐min vortex and incubation at −20°C for 2 h, the samples were centrifuged at 4000 rpm for 20 min, and then the supernatant was transferred to vacuum freeze‐drying. Metabolites were resuspended in 150 μL of 50% methanol, centrifuged at 4000 rpm for 30 min, and the supernatants were transferred to liquid chromatography–mass spectrometry (LC–MS) analysis.^35^, ^36^ Raw data were imported into Compound Discoverer 3.1 (Thermo Fisher Scientific, Waltham, Massachusetts) and MetaX for further processing.

### 2.7 Bioinformatics and statistical analysis

For microbiome analysis, Venn plots of ASVs were performed by the “Venn diagram” package in R. Nonmetric multidimensional scaling (NMDS), principal coordinate analysis (PCoA) were used to analyze the β‐diversity. The Wilcoxon rank sum test was used to compare α‐diversity and bacterial communities among the three groups, and α‐diversity, including Shannon, Simpson, Chao1, and abundance‐based coverage estimator (Ace), was estimated by Mothur 1.31.2. Furthermore, linear discriminant analysis effect size (LefSe) was used to identify altered microbiota with the linear discriminant analysis (LDA) score >2.0.^37^ To further determine microbial biomarkers that can differentiate between TD− and TD+, a random forest (RF) model was constructed using the area under the curve (AUC)‐RF algorithm.^38^ The input variable for the classification model was only included if it was present in more than 20% of samples and its relative abundance was greater than 0.05%. The RF model was established by receiver operating characteristic (ROC) curve analysis.^39^

For metabolomics analysis, the unsupervised principal component analysis (PCA) and supervised orthogonal partial least squares discriminant analysis (OPLS‐DA) were conducted to determine the distributions and identify the metabolic difference in the two groups. The screening conditions for the differential metabolites were as follows: (1) fold change (FC) ≥1.20 or ≤0.83, (2) *p* < 0.05, and (3) variable importance in projection (VIP) value ≥1.0. The metabolites were determined with a combination of the Kyoto Encyclopedia of Genes and Genomes (KEGG) database.^40^

Data were expressed as the mean ± SD for continuous variables, and frequency and percentage (%) were used for categorical variables. Comparisons between different groups were analyzed by *t* test or nonparametric test. To analyze the correlation network in microbiota, Spearman's correlation coefficients among genera were calculated. Correlations with an absolute coefficient value ≥0.6 and *p* value <0.05 were transformed into associations between two genera. Network graphs were constructed by Cytoscape 3.9.1. Spearman's rank correlation was also conducted to show the correlation among microbiota, metabolites, and clinical parameters. Pairwise comparisons of environmental factors were shown by the Mantel test, with a color gradient indicating Spearman's correlation coefficients. A *p* value <0.05 was considered to be statistically significant. All statistical analyses were performed using SPSS version 23.0 software (IBM Corporation, New York), GraphPad Prism 8.4.3 software (GraphPad Software Inc., San Diego, California), and the statistical program R (version 4.0.2; The R Foundation, Vienna, Austria).

## 3 RESULTS

### 3.1 Clinical characteristics of study population

The clinical characteristics of the study population are presented in Table 1. There were no significant differences in sex, age, duration, body mass index (BMI), waist‐to‐hip ratio (WHR), glycosylated hemoglobin (HbA1c), fasting C‐peptide (FCP), total cholesterol (TC), triglyceride (TG), high‐density lipoprotein cholesterol (HDL‐C) and low‐density lipoprotein cholesterol (LDL‐C) between patients in TD− and TD+. However, patients in TD+ had a higher insulin dose (0.77 ± 0.23 u/kg vs. 0.64 ± 0.20 u/kg for TD+ and TD− group, respectively; *p* = 0.018) and fasting plasma glucose (FPG) (9.19 ± 3.86 mmol/L vs. 7.29 ± 2.39 mmol/L for TD+ and TD− group, respectively; *p* = 0.015) than those in TD−.

**TABLE 1:** Demographic and clinical characteristics of participants.

		T1D (*n* = 72)		
Variable	HC (*n* = 29)	TD− (*n* = 35)	TD+ (*n* = 37)	*p* value
Sex (female, %)	19 (65.52%)	20 (57.14%)	23 (62.16%)	0.664
Age (years)	31.76 ± 7.99	32.49 ± 8.48	32.65 ± 8.11	0.934
Duration (years)	‐	12.89 ± 5.81	11.85 ± 6.00	0.458
Insulin dose (u/kg)	‐	0.64 ± 0.20	0.77 ± 0.23	0.018
BMI (kg/m^2^)	21.98 ± 2.91	21.18 ± 1.81	21.36 ± 2.20	0.703
WHR	0.81 ± 0.08	0.80 ± 0.06	0.82 ± 0.06	0.120
HbA1c (%)	5.20 ± 0.32	6.94 ± 1.13	7.53 ± 1.60	0.077
FPG (mmol/L)	4.47 ± 0.44	7.29 ± 2.39	9.19 ± 3.86	0.015
FCP (nmol/L)	0.35 ± 0.11	0.04 ± 0.05	0.05 ± 0.06	0.499
TC (mmol/L)	4.74 ± 0.73	4.66 ± 0.71	4.98 ± 0.93	0.104
TG (mmol/L)	1.00 ± 0.56	0.74 ± 0.33	0.81 ± 0.35	0.343
HDL‐C (mmol/L)	1.28 ± 0.24	1.52 ± 0.24	1.58 ± 0.36	0.461
LDL‐C (mmol/L)	2.83 ± 0.67	2.77 ± 0.76	3.05 ± 0.68	0.102
Depression scores	38.58 ± 7.89	45.71 ± 4.30	56.35 ± 3.23	<0.001

*Note*: Data are mean (SD) or *n* (%). *p* values were compared by the two groups (TD−; TD+).

Abbreviations: BMI, body mass index; FCP, fasting C‐peptide; FPG, fasting plasma glucose; HC, healthy control; HbA1c, glycosylated hemoglobin; HDL‐C, high‐density lipoprotein cholesterol; LDL‐C, low‐density lipoprotein cholesterol; T1D, type 1 diabetes; TC, total cholesterol; TD−, T1D patients without depression; TD+, T1D patients with depression; TG, triglyceride; WHR, waist‐to‐hip ratio.

### 3.2 Bacterial diversity among HC, TD−, and TD+

**FIGURE 2:**
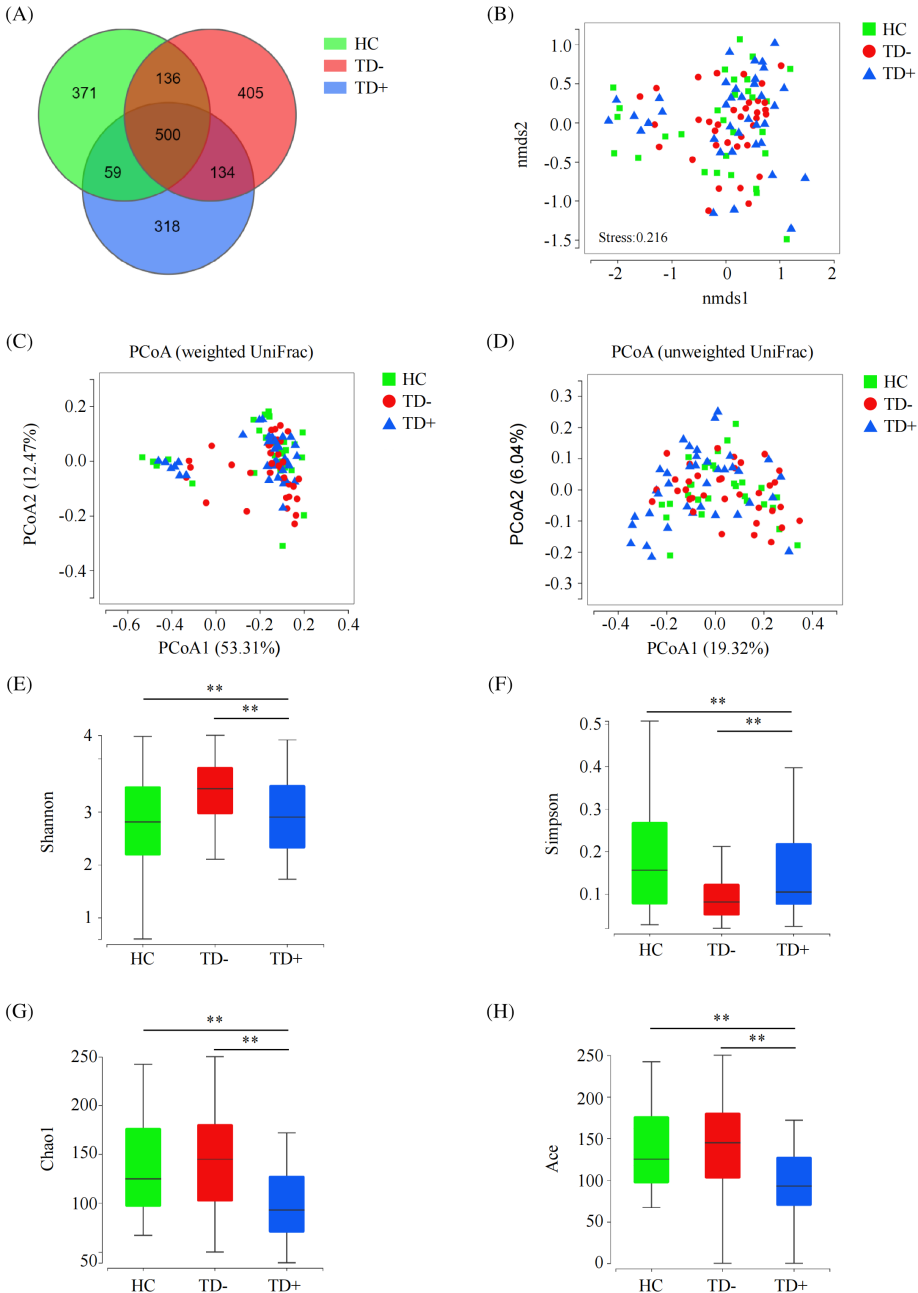
Alpha and Beta diversity among HC, TD− and TD+. (A) The Venn diagram among HC, TD− and TD+; (B) NMDS analysis of β‐diversity based on the Bray–Curtis dissimilarity; (C, D) PCoA analyses based on weighted (left) and unweighted (right) UniFrac distances in HC, TD− and TD+; (E–H) Box plots of differences in α‐diversity of the gut microbiota of Shannon (E), Simpson (F), Chao1 (G) and Ace (H) indices. *: *p* < 0.05; **: *p* < 0.01. Ace, abundance‐based coverage estimator; HC, healthy controls; NMDS, non‐metric multidimensional scaling; PCoA, principal co‐ordinates analysis; TD−, T1D patients without depression; TD+, T1D patients with depression.

A total of 7 525 573 high‐quality reads (2 151 588 reads in HC, 2611588 reads in TD−, and 2 762 397 reads in TD+) with an average of 74 511 reads per sample were obtained for microbiota analysis. Deeper sequencing identified the majority of the bacterial phylotypes (1552 ASVs) present in T1D patients. Based on the Venn diagram, we observed a slightly lower number of ASVs in TD+ (Figure 2A). In β‐diversity analyses, compared to NMDS analysis and PCoA based on weighted UniFrac distances, PCoA conducted based on unweighted UniFrac distances could more effectively separate the three groups into distinct clusters (Pr [>*F*] < 0.001) (Figure 2B–D). Regarding bacterial α‐diversity analyses, we observed significant differences in the Shannon, Simpson, Chao1, and Ace indices among the HC, TD−, and TD+ groups (*p* < 0.05), where TD+ exhibited decreased diversity compared to TD− and HC (*p* < 0.05) (Figure 2E–H).

### 3.3 Altered fecal microbiota composition among HC, TD−, and TD+

**FIGURE 3:**
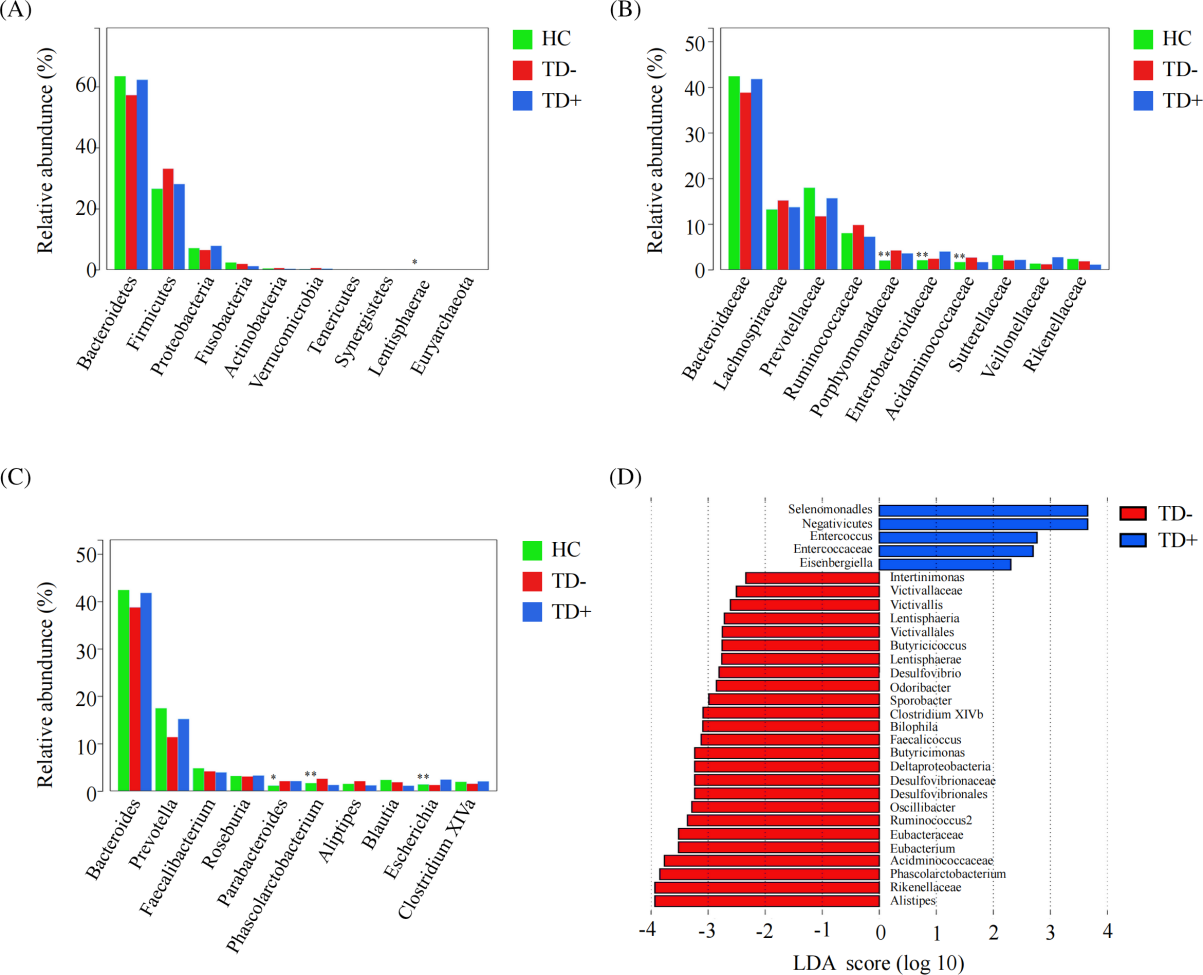
Microbiotic characteristics among HC, TD− and TD+. (A–C) Top 10 abundant gut microbiota at the phyla (A), family (B) and genera (C) level among HC, TD− and TD+; (D) The bacterial phylotypes between TD− and TD+ based on LefSe analysis. *: *p* < 0.05; **: *p* < 0.01. HC, healthy controls; LefSe, linear discriminant analysis effect size; TD−, T1D patients without depression; TD+, T1D patients with depression.

The overall microbial compositions in the HC, TD−, and TD+ groups were examined at different taxonomic levels. The top 10 most abundant microbiota at the phylum, family, and genus levels among the three groups are shown in Figure 3A–C. We revealed significant differences in the relative abundance of Lentisphaerae (phylum), Porphyromonadaceae (family), Enterobacteriaceae (family), Acidaminococcaceae (family), *Parabacteroides* (genus), *Phascolarctobacterium* (genus), and *Escherichia* (genus) among the HC, TD−, and TD+ groups (*<p* < 0.05). LEfSe identified many bacterial phylotypes that could potentially distinguish TD+ from TD− (Figure 3D). At the phylum level, Lentisphaerae was observed to differ significantly between the two groups. At the family level, we found Enterococcaceae were enriched in TD+, while Eubacteriaceae, Victivallaceae, Acidaminococcaceae, Rikenellaceae, and Desulfovibrionaceae were significantly decreased. At the genus level, *Phascolarctobacterium*, *Ruminococcus2*, *Bilophila*, *Butyricicoccus*, *Clostridium* XlVb, *Alistipes*, *Intestinimonas*, *Oscillibacter*, *Odoribacter*, *Sporobacter*, *Butyricimonas*, *Victivallis*, *Desulfovibrio*, *Eubacterium*, and *Faecalicoccus* were significantly enriched in TD−, while *Eisenbergiella* and *Enterococcus* were markedly reduced. Together, these results revealed differences in microbial diversity and structure between TD− and TD+.

### 3.4 Multivariate analyses and metabolites identification between TD− and TD+

**FIGURE 4:**
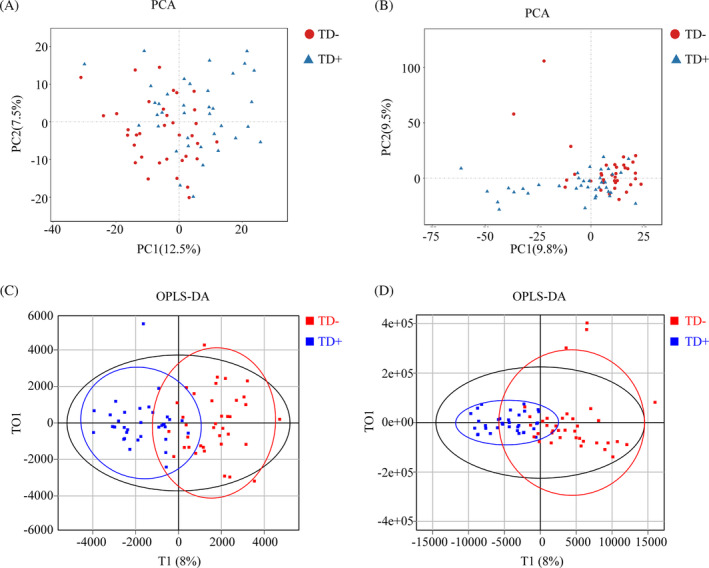
Metabolic characteristics between TD− and TD+. (A, B) The score plots of PCA models in positive‐ion mode (A) and negative‐ion mode (B) between TD− and TD+;<(C, D) The score plots of OPLS‐DA models in positive‐ion mode (C) and negative‐ion mode (D) between TD− and TD+.

The distribution of samples was determined by PCA. As shown in the score plot in positive or negative ion modes, TD− and TD+ were not separated (Figure 4A, B). To optimize the separation of the two groups, OPLS‐DA was used to visualize their metabolic differences. The score plots of OPLS‐DA were shown in Figure 4C, D. Following the response test of score plots, no significant evidence of complete over‐fitting was observed, indicating the construction of a good OPLS‐DA model. These results showed that TD− and TD+ had different metabolic characteristics. Differential metabolites were further screened based on the VIP values obtained from the positive and negative OPLS‐DA models. A total of 54 metabolites (40 upregulated and 14 downregulated metabolites) in TD+ versus TD− were identified, and 14 differential metabolites (11 upregulated in TD+ [including 4‐methylene‐2‐oxoglutarate; 4‐tert‐octylphenol; di‐isodecyl phthalate; 12,13‐DHOME, 11‐aminoundecanoic acid; glutaric acid; bis (2‐ethylhexyl) phthalate; allamandin; diethyl pyrocarbonate; soyasapogenol A; and malondialdehyde] and 3 downregulated metabolites in TD+ [including polygodial, allopregnanolone, and uric acid]) were mapped to KEGG (Table 2).

**TABLE 2:** Metabolite identification in serum from T1D patients with depression versus without depression.

Compound name	ESI^+/−^	Formula	KEGG ID	FC	VIP	*p* value	Trend in TD+
Polygodial	+	C_15_H_22_O_2_	C09712	0.81	1.90	0.03	Down
Allopregnanolone	+	C_21_H_34_O_2_	C13712	0.81	1.55	0.02	Down
Uric acid	+	C_5_H_4_N_4_O_3_	C00366	0.83	4.37	<0.01	Down
4‐methylene‐2‐oxoglutarate	+	C_6_H_6_O_5_	C06035	1.32	2.02	0.01	Up
4‐tert‐octylphenol	+	C_14_H_22_O	C14205	1.30	1.02	0.02	Up
Di‐isodecyl phthalate	+	C_28_H_46_O_4_	C14578	1.23	2.05	0.01	Up
12,13‐DHOME	+	C_18_H_34_O_4_	C14829	1.28	2.03	0.01	Up
11‐aminoundecanoic acid	+	C_11_H_23_NO_2_	C19325	1.44	2.66	<0.01	Up
Glutaric acid	−	C_5_H_8_O_4_	C00489	1.22	2.10	0.01	Up
Bis (2‐ethylhexyl) phthalate	−	C_24_H_38_O_4_	C03690	1.28	1.12	0.01	Up
Allamandin	−	C_15_H_16_O_7_	C09766	1.97	1.67	0.02	Up
Diethyl pyrocarbonate	−	C_6_H_10_O_5_	C11592	1.24	1.95	0.02	Up
Soyasapogenol A	−	C_30_H_50_O_4_	C17419	1.30	1.08	0.01	Up
Malondialdehyde	−	C_3_H_4_O_2_	C19440	1.23	1.42	0.02	Up

Abbreviations: ESI, electron spray ionization; FC, fold change; KEGG, Kyoto Encyclopedia of Genes and Genomes; T1D, type 1 diabetes; TD+, T1D patients with depression; VIP, variable importance in projection.

### 3.5 Correlation analysis of altered microbiota, metabolites, and clinical characteristics between TD− and TD+

**FIGURE 5:**
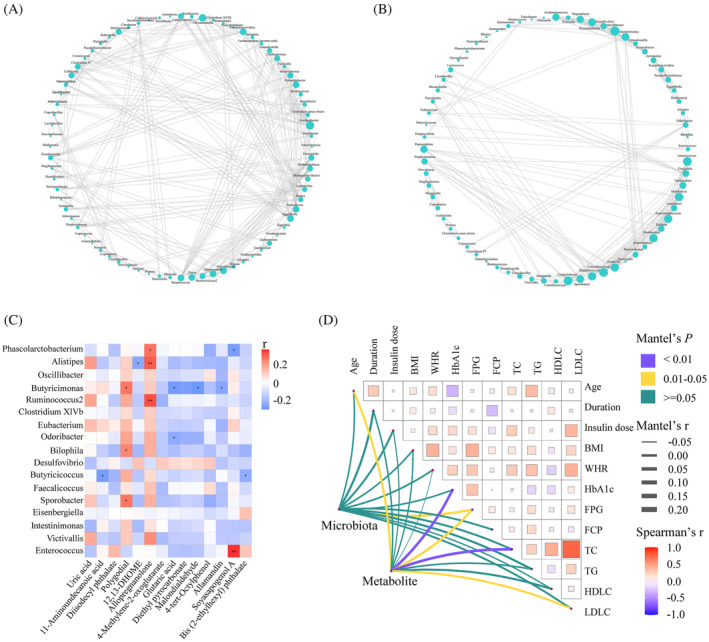
Correlation analysis of altered gut microbiota, serum metabolites and clinical characteristics between TD− and TD+. (A, B) The correlation network at the genera level of microbiota in TD− (A) and TD+ (B). The correlation coefficient was calculated with Spearman's rank correlation test (|*r*| ≥ 0.6); (C) The correlation heatmap of the altered fecal microbiota and serum metabolites between TD− and TD+; (D) The clinical characteristics correlated with altered fecal microbiota and serum metabolites between TD− and TD+ based on the Mantel test. BMI, body mass index; FCP, fasting C‐peptide; FPG, fasting plasma glucose; HbA_1c_, glycosylated hemoglobin; HDLC, high‐density lipoprotein cholesterol; LDLC, low‐density lipoprotein cholesterol; TC, total cholesterol; TG, triglyceride; TD−, T1D patients without depression; TD+, T1D patients with depression; WHR, waist‐to‐hip ratio.

The co‐occurrence network between TD− and TD+ was totally different (Figure 5A,B). The network was simplified in TD+ with a reduced number of nodes. More fragmented subnetworks appeared in TD+ due to the lower clustering coefficient of the network. Then we constructed a correlation heatmap to explore associations of altered microbiota and metabolites (Figure 5C). *Butyricimonas* was positively correlated with polygodial but negatively correlated with three metabolites (glutaric acid, malondialdehyde, and allamandin); *Phascolarctobacterium* was positively correlated with allopregnanolone but negatively correlated with soyasapogenol A; *Alistipes* was positively correlated with allopregnanolone but negatively correlated with 12,13‐DHOME; *Butyricicoccus* was negatively correlated with 11‐aminoundecanoic acid and bis (2‐ethylhexyl) phthalate. We also found that *Bilophila* and *Sporobacter* were positively correlated with polygodial. Additionally, to identify the impact of environmental factors, we correlated altered microbiota (*Phascolarctobacterium*, *Ruminococcus2*, *Bilophila*, *Butyricicoccus*, *Clostridium* XlVb, *Alistipes*, *Intestinimonas*, *Oscillibacter*, *Odoribacter*, *Sporobacter*, *Butyricimonas*, *Victivallis*, *Desulfovibrio*, *Eubacterium*, *Faecalicoccus*, *Eisenbergiella*, and *Enterococcus*) and metabolites (4‐methylene‐2‐oxoglutarate; 4‐tert‐octylphenol; di‐isodecyl phthalate; 12,13‐DHOME; 11‐aminoundecanoic acid; glutaric acid; bis (2‐ethylhexyl) phthalate; allamandin; diethyl pyrocarbonate; soyasapogenol A; malondialdehyde; polygodial; allopregnanolone; and uric acid) with clinical characteristics (including age, duration, insulin dose, BMI, WHR, HbA1c, FPG, FCP, TC, TG, HDL‐C, and LDL‐C). Based on the Mantel test, we found that FPG was the common correlate of both altered microbiota and metabolites (Figure 5D).

### 3.6 Microbial signature associated with TD+

**FIGURE 6:**
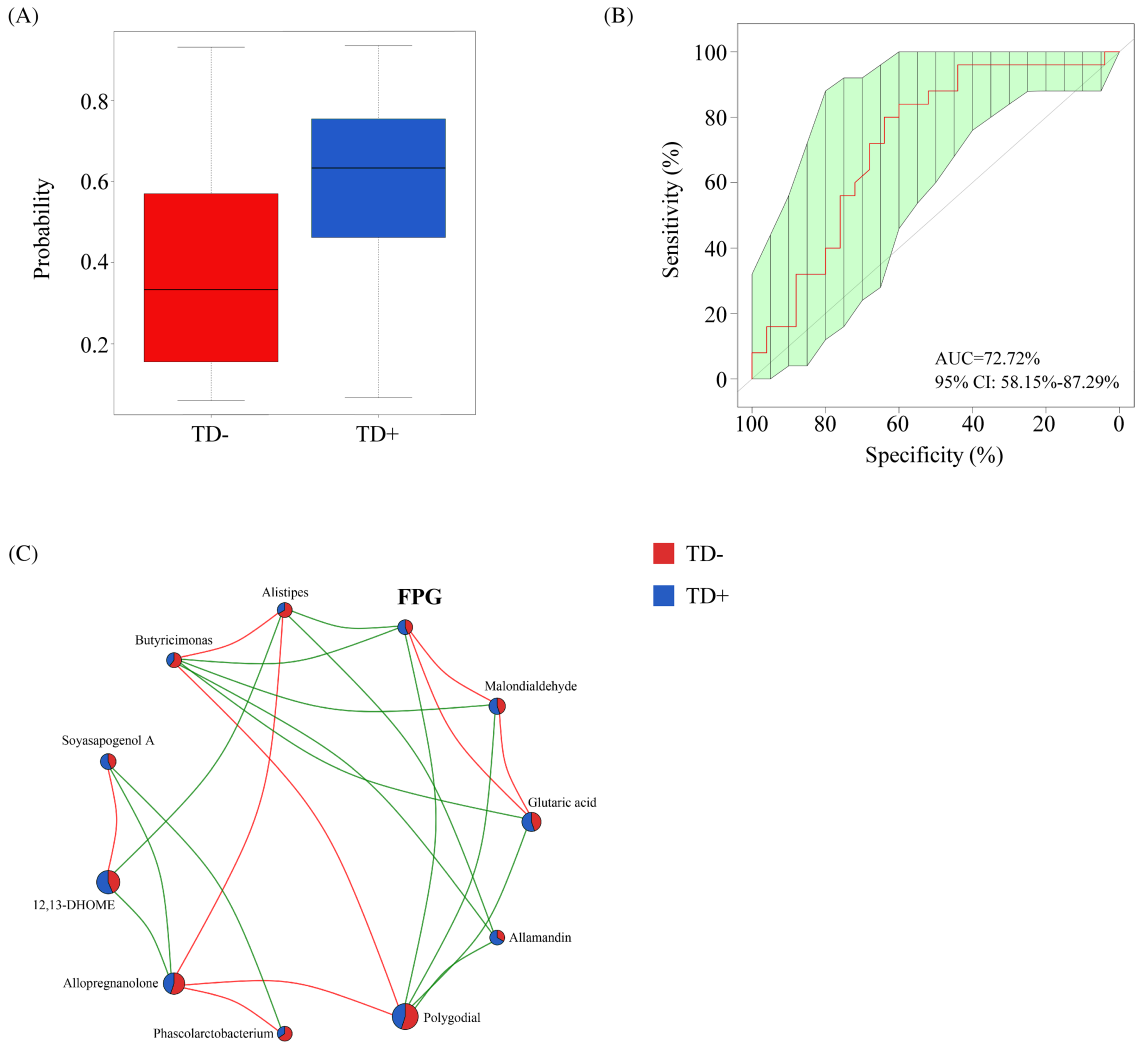
Identification of microbial signature associated with TD+. (A) The probability value of RF model between TD− and TD+; (B) The ROC curve of the optimal model for classifying TD+ from TD−; (C) The correlation among key microbiota, their related metabolites and FPG. FPG, fasting plasma glucose; TD−, T1D patients without depression; TD+, T1D patients with depression; RF, random forest; receiver operating characteristic.

To further identify microbial signatures that can differentiate TD+ from TD−, an RF classifying model was constructed using the AUC‐RF algorithm. The results demonstrated a minimal set of five genera that maximally distinguished TD− from TD+. It contained *Phascolarctobacterium*, *Butyricimonas*, *Alistipes*, *Lachnospiracea incertae sedis*, and *Blautia*. The probability value of this training model in TD+ increased significantly compared with that in TD− (Figure 6A), and the AUC value was 0.73 (95% CI, 0.58–0.87) (Figure 6B). Most of these (including *Phascolarctobacterium*, *Butyricimonas*, and *Alistipes*) had an LDA value >2.0, which significantly reduced in TD+. We also explored the relationship among these microbiota, their related metabolites, and FPG (Figure 6C). FPG was negatively correlated with *Butyricimonas* (*r* = −0.40, *p* < 0.01) and its related metabolites (polygodial [*r* = −0.25, *p* = 0.03], glutaric acid [*r* = 0.55, *p* < 0.01], and malondialdehyde [*r* = 0.56, *p* < 0.01]).

## 4 DISCUSSION

In the present study, we demonstrated the profile of the gut microbiota and serum metabolites in T1D with depression through high‐throughput sequencing technology, and constructed the relationship among microbiota, metabolites, and clinical characteristics.

Compared to those without mental disorders, patients with depression and diabetes had more serious complications and higher mortality.^41^ Both depression and diabetes are connected with alterations in metabolic pathways and stress networks, leading to disorders of blood glucose and insulin resistance.^42^, ^43^ In this study, although the difference was not statistically significant, HbA1c was 0.59% higher in TD+ than TD−. Moreover, TD+ had higher FPG and daily insulin doses, which suggested that depression might play a significant role in adherence to diabetes care and result in poor glycemic control.

Growing evidence in recent years has established the role of gut microbiota in depression. Currently, there are few studies that identified the microbial signature in T1D with depression. Petrak et al. found that the composition of the gut microbiota was associated with the development of depression in T1D, and no difference in α‐diversity was observed between T1D with depression and T1D without depression, while T1D with depression had a higher abundance of *Megasphaera*.^25^ These findings hint at a potential link between specific bacterial groups and depression in people with diabetes. In our study, the Shannon, Chao1, and Ace indices of TD− were significantly higher than those of TD+, suggesting that TD+ had a lower α‐diversity of gut microbiota. Consistent with our results, previous studies have found that α‐diversity is often lower in patients with depression than in controls.^44^, ^45^ Furthermore, the imbalance of microbiota was mainly reflected by changes at the genus level. We found 15 genera enriched in TD− and 2 genera enriched in TD+ by LefSe analysis, and most of them (*Phascolarctobacterium*,^46^, ^48^, ^49^, ^51^, ^53^ *Bilophila*,^50^ *Butyricicoccus*,^47^ *Clostridium* XlVb,^46^ *Alistipes*,^46^, ^50^, ^52^, ^53^, ^54^ *Intestinimonas*,^53^ *Oscillibacter*,^46^, ^49^, ^52^ *Odoribacter*,^54^ *Sporobacter*,^51^ *Butyricimonas*,^46^, ^48^ *Desulfovibrio*,^48^ *Eubacterium*,^48^, ^49^ and *Enterococcus* ^49^, ^52^) have been reported in human studies on depression. We generated an RF model for predicting T1D with depression and showed high predictive accuracy (AUC = 0.73). And the majority (3/5, including *Phascolarctobacterium*, *Butyricimonas*, and *Alistipes*) of key bacteria identified by RF analyses were contained in the above 17 genera. In addition, metabolite changes between groups were distinguished based on the OPLS‐DA model. In the present study, a total of 14 differential metabolites mapped to KEGG were identified. Among them, allopregnanolone,^55^, ^56^ uric acid,^57^, ^58^ glutaric acid,^59^, ^60^ bis (2‐ethylhexyl) phthalate,^61^ and malondialdehyde^62^, ^63^ have been reported in previous studies on depression. In these studies, allopregnanolone was found to be involved in steroid hormone metabolism,^55^ uric acid in purine metabolism,^58^ glutaric acid in lysine and fatty acid metabolism,^59^, ^60^ bis (2‐ethylhexyl) phthalate in glutamate metabolism,^61^ and malondialdehyde in lipoprotein metabolism.^62^

As stated above, we discovered a simplified network in TD+ in comparison with TD−. Bacteria coexist in complex interaction webs, and perturbations within these webs may contribute to disease.^64^ Our finding suggested the loss of certain microbial interactions in TD+, leading to destruction of the homeostasis of the microbiota. Furthermore, the production of metabolites by microbes contributes to the host metabolic phenotype and influences the disease risk. Here, we explored the relationship between key bacteria and differential metabolites. *Butyricimonas* was negatively correlated with glutaric acid and malondialdehyde. *Butyricimonas* is a butyrate producer, which contributes to intestine epithelial integrity.^65^ Decreased levels of *Butyricimonas* were found in T1D and depression.^11^, ^46^ Glutaric acid is produced during the metabolism of lysine. Fluctuations in blood glucose levels could affect this metabolic pathway.^66^ Lu et al. found that the level of glutaric acid was higher in patients with fulminant T1D than in HCs.^67^ Glutaric acid was also identified as a potential biomarker of chronic unpredictable mild stress (CUMS)‐induced depression.^59^ Patients with postpartum depression had higher levels of glutaric acid than those without.^60^ Malondialdehyde, the final product of polyunsaturated fatty acid peroxidation in the cells, is commonly known as a marker of oxidative stress. Elevated malondialdehyde levels have been observed in individuals with T1D, and malondialdehyde can further contribute to cellular damage and impair insulin sensitivity, exacerbating the progression of the disease.^68^, ^69^ In recent years, oxidative stress has received much attention with regard to psychiatric illnesses. Malondialdehyde was found to be elevated in patients with major depression and depressed patients with physical ailments, such as gastric adenocarcinoma, chronic heart failure, and stroke.^62^, ^70^, ^71^

Furthermore, our results suggested that both *Phascolarctobacterium* and *Alistipes* were positively correlated with allopregnanolone. *Phascolarctobacterium* produces short‐chain fatty acids (including acetate and propionate) and is involved in energy metabolism and immune inflammation.^72^ The levels of *Phascolarctobacterium* were higher in healthy subjects than T1D patients.^73^ On the other hand, Humbel et al. found that *Phascolarctobacterium* was negatively correlated with depressive symptoms in major depression.^48^ *Alistipes*, a relatively new genus of bacteria isolated from clinical samples, may promote mucous production and a healthy intestinal epithelial barrier in T1D.^74^ However, Ma et al. found that *Alistipes* was upregulated in T1D rats injected with streptozotocin.^75^ Evidence for the involvement of *Alistipes* in depression is also controversial. Caso et al. reported enrichment of the *Alistipes* in the depressed patients, but Zhang et al. found that *Alistipes* was relatively more abundant in the HCs compared to patients with major depressive disorder.^50^, ^52^ Allopregnanolone is a naturally occurring neurosteroid from the hormone progesterone and a positive allosteric modulator of γ‐aminobutyric acid (GABA).^76^ It has been shown to have anti‐inflammatory and immunomodulatory effects, which could potentially help protect against cognitive deficit and dysbiosis in T1D.^77^ And reduced levels of allopregnanolone in the peripheral blood or cerebrospinal fluid were found to be associated with depression.^78^

In addition, our study discovered the relationship among key microbiota, their related metabolites, and FPG. These results suggested that *Butyricimonas* might affect FPG in T1D patients with depression through the glutaric acid and malondialdehyde metabolic pathways. In the future, we will explore their causal relationship in T1D with depression and investigate the biological mechanism through further study.

There are some limitations to our study. Firstly, this study included a relatively small sample size which was insufficient to completely reflect microbial and metabolic changes in T1D with depression. Secondly, all participants were recruited from the same site and were of Han ethnicity. Thus, site‐specific and ethnic biases in microbial phenotypes could not be ruled out. Thirdly, we used the 16S RNA sequencing method to assess the gut microbiota, which might not be as accurate for those species. Fourthly, it would be more scientifically rigorous to set up a separate control group for patients with depression but without T1D. The interdisciplinary collaboration between endocrinology and psychiatry is expected in the future.

In conclusion, our study indicated significant alterations in gut microbiota and serum metabolites in T1D with depression. T1D patients with depression were characterized by unique profiles of gut microbiota and serum metabolites. *Phascolarctobacterium*, *Butyricimonas*, and *Alistipes* could predict the risk of T1D with depression. These findings provide further evidence that the microbiota–gut–brain axis is involved in T1D with depression.

## AUTHOR CONTRIBUTIONS

Conception and design of the study: X.Z., S.L., W.X., J.Y., J.W., D.Y., and C.W. Acquisition of data was performed by Z.L., X.Z., and S.L. Collection, analysis, and interpretation of data: Z.L., T.Y., S.L., and C.W. Preparation of figures and tables and writing of original draft: Z.L., D.Y., and C.W. All authors contributed to the article and approved the submitted version.

## FUNDING INFORMATION

This study was supported by the National Key R&D Program of China (grant no. 2017YFC1309600), the Guangdong Basic and Applied Basic Research Foundation (grant no. 2019A1515010979), the National Natural Science Foundation of China (grant no. 82100822), the Anhui Provincial Natural Science Foundation (grant no. 2008085MH248 & 2008085MH278), and the Science and Technology Projects in Guangzhou (grant no. 2023A04J1087).

## DISCLOSURE

The authors declare that they have no conflict of interest.
